# Les accidents de cyclomoteurs: mécanismes lésionnels et aspects anatomo-cliniques

**DOI:** 10.11604/pamj.2015.21.332.6651

**Published:** 2015-08-31

**Authors:** Thomas Marcel Mbar Wade, Papa Abdoulaye Ba, Mamadou Moustapha Niane, Malick Cissé N'diaye, Ibrahima Konaté, Cheikh Tidiane Touré

**Affiliations:** 1 Service de Chirurgie Générale, Centre Hospitalier Régional de Kafrrine, Sénégal; 2 Département de Chirurgie, UFR Santé, Université de Thiès, Sénégal; 3 Région Médicale de Kaffrine, Sénégal; 4 Clinique Chirurgicale CHU Aristide Le Dantec, Dakar, Sénégal

**Keywords:** Cyclomoteur, lésion, mécanisme, clinique, moped, lesion, mecanism, clinique

## Abstract

Le but de notre étude est de décrire les mécanismes lésionnels et les aspects anatomo-cliniques des traumatismes par accident de cyclomoteur. C'est une étude transversale menée au niveau du Centre Hospitalier Régional de Kaffrine sur une période de 12 mois. Elle portait sur les patients admis au service d'accueil pour accident de la voie publique impliquant un cyclomoteur. Il s'agissait de 129 patients (112 hommes et de 17 femmes). L’âge moyen était de 30,5 ans. Soixante-treize patients étaient conducteurs de cyclomoteur, 31 piétons et 25 passagers arrière. Le mécanisme le plus fréquent était une chute de moto. Les lésions prédominaient au niveau des membres. Les accidents de cyclomoteur sont un problème de santé publique.

## Introduction

Les accidents des cyclomoteurs posent un problème de santé publique [1]. Ils constituent 50% de l'ensemble des blessés ou tués sur les routes [2]. L'avènement de nouveaux engins plus puissants et financièrement plus accessibles accentue le problème en Afrique. A Kaffrine, ville du centre du Sénégal de faible agglomération, les accidents de cyclomoteurs constituent l'un des motifs de consultation les plus fréquents au service d'Accueil du Centre Hospitalier Régional. Nous avons mené au niveau de cet hôpital une étude transversale dont le but était de décrire les mécanismes lésionnels et les aspects anatomo-cliniques des traumatismes par accident de cyclomoteur.

## Méthodes

C'est une étude transversale menée sur une période de 12 mois (1er juillet 2011 au 30 juin 2012) au niveau du Centre Hospitalier Régional de Kaffrine. Elle portait sur les patients admis au service d'Accueil pour accident de la voie publique ayant impliqué un cyclomoteur. Durant la période d’étude, 223 accidentés de la voie publique étaient reçus. Cent vingt-neuf répondaient à nos critères d'inclusion. Le mécanisme de l'accident était précisé à l'interrogatoire. Le bilan lésionnel basé sur les examens clinique et paraclinique était décrit en fonction du mécanisme de l'accident. Les séquelles étaient évaluées après une année de suivi des patients. Les paramètres étudiés étaient les aspects épidémiologiques, les circonstances et mécanismes de l'accident et le bilan lésionnel en fonction du mécanisme.

## Résultats

### Aspects épidémiologiques

**Figure 1:**
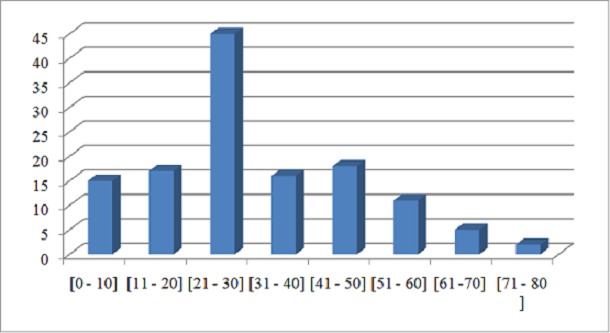
Répartition des patients selon la tranche d’âge

Il s'agissait de 129 patients dont 112 hommes (86,8% des cas) et de 17 femmes (13,2% des cas). L’âge moyen était de 30,5 ans avec des extrêmes de 2 ans et 75 ans. La tranche d’âge 21-30 ans était la plus représentative avec un effectif de 45 cas (Figure 1).

### Circonstances et mécanismes de l'accident

L'accident avait lieu en milieu urbain dans 87cas (67,4%), sur une route secondaire bitumée dans 28 cas (21,7%) et sur une route nationale dans 14 cas (10,9%). Les patients étaient des conducteurs dans 73 cas (56,6%), des piétons dans 31 cas (24%) et des passagers arrière dans 25 cas (19,4%). Seuls 6 patients portaient un casque (4,7% des cas) et 2 une tenue de protection (1,6% des cas).

Le mécanisme était: une chute dans 55 cas (42,7%) due à: une perte d'adhérence dans 28 cas, une manoeuvre d’évitement dans 12 cas, un mauvais état de la route dans 11 cas, un problème mécanique de l'engin dans 4 cas; une collision dans 43 cas (33,3%) avec: un cyclomoteur dans 21 cas, une charrette dans 11 cas, un véhicule léger dans 10 cas et un véhicule lourd dans un cas; un renversement de piéton par un cyclomoteur dans 31 cas (24%).

### Bilan lésionnel selon le mécanisme

Les lésions prédominaient au niveau des membres (Tableau 1). Les lésions cutanéo-muqueuses (dermabrasions, brûlures et plaies des parties molles) étaient les plus fréquentes (n = 52) suivies par les fractures de membre (n = 24). Chez les piétons les lésions dominaient au niveau des membres inférieurs. Le Tableau 2 résume le bilan lésionnel en fonction du mécanisme.

**Tableau 1:** Fréquence des urgences selon le siège de la lésion

Siège de la lésion	Nombre	Pourcentage
Membres inférieurs	64	49,6
Membres supérieurs	34	26,4
Crâne	10	7,8
Thorax	9	7
Bassin	4	3,1
Visage	3	2,3
Abdomen	2	1,6
Bouche	2	1,6
Rachis	1	0,8
**TOTAL**	**129**	**100**

**Tableau 2:** Bilan lésionnel en fonction du mécanisme

Type de lésion	Chute	Collision				Piétons	Total
		Moto	vl	VL	Charrette		
Fracture fermée de membre	10	3	4		1	2	**20**
Fracture ouverte de membre	1					3	**4**
Entorse	10	4	3			3	**20**
Luxation	2						**2**
Amputation orteil	2						**2**
Fracture du bassin	2					2	**4**
Contusion abdominale					2		**2**
Traumatisme thorax	2	2	1		3	1	**9**
TCE	2	1			2	5	**10**
Fracture rachis cervical				1			**1**
Délabrement de membre		2				1	**3**
Cutanéo-muqueuses	24	9	2		3	14	**52**
**Total**	**55**	**21**	**10**	**1**	**11**	**31**	**129**

Trois patients étaient décédés sur le lieu de l'accident et trois autres dans les suites précoces de l'accident, soit une mortalité de 4,6% (n = 6). Les mécanismes des décès étaient une collision avec une charrette dans 4 cas, une collision avec un véhicule lourd dans un cas et un renversement de piéton par un cyclomoteur dans un cas. Les causes de décès étaient un traumatisme du thorax dans 3 cas, de l'abdomen dans un cas, du rachis cervical dans un cas et un choc anaphylactique chez un patient qui présentait une fracture ouverte de jambe.

## Discussion

Les accidents de cyclomoteurs constituent un problème de santé publique [1]. Ils représentent 57,8% des cas dans notre étude. Ces accidents sont en constante augmentation dans nos régions du fait de la prolifération d'engins de grande vitesse, du mauvais état des routes et du non-respect des règles élémentaires du code de la route, [3–5]. Aussi l'utilisation de la chaussée par les véhicules hippomobiles augmente le risque encouru par les motocyclistes comparativement aux automobilistes. En effet, dans notre série 4 des 6 décès étaient survenus après une collision avec une charrette. Les sujets jeunes du sexe masculin sont les plus concernés par ces accidents [3, 6]. Signe de liberté et de rapidité de déplacement, les deux-roues motorisés ont séduit beaucoup de jeunes garçons. Le milieu urbain est fortement concerné avec 67,4% des cas dans notre série.

Le mécanisme le plus fréquent dans notre étude est la chute de moto. Dans les grandes agglomérations africaines par contre, la collision est le mécanisme le plus fréquent [5]. N’étant pas intrinsèquement stable, un deux-roues motorisé peut chuter seul par défaut de la route, perte d'adhérence, manoeuvre d’évitement d'un obstacle ou d'un véhicule, malaise du conducteur ou son état défectueux [7]. En cas de chute on s'attend aux traumatismes habituels des chutes de faible hauteur, amplifiés par la vitesse, auxquels viennent s'ajouter les traumatismes primaires résultant de la percussion de l'engin ou de l’écrasement par l'engin. Si les pieds touchent le sol lorsque l'engin roule, on a d'importantes lésions du pied avec risque d'amputation traumatique d'orteil. C’était le cas chez deux de nos patients. En cas de collision, le cyclomotoriste est projeté sur le véhicule avec risque de fractures de vertèbres cervicales par hyper-extension du cou. Les lésions vertébrales peuvent aussi se produire lors de l'impact sur le sol. Chez un de nos patients, la lésion du rachis cervical était due à une hyper-extension suite à sa projection sur un véhicule lourd. Une collision avec un autre cyclomoteur provoque le plus souvent des traumatismes des membres qui sont exposés lors du contact. S'il s'agit d'un piéton renversé par un cyclomoteur, il peut présenter des lésions dues au choc avec la moto ou être projeté sur le sol. Les dégâts sont en général d′autant plus importants que l'engin est rapide et massif. Le siège et le degré de la lésion dépendent de la taille du piéton et de la vitesse de l'engin: les enfants présentant des traumatismes du tronc et crâniens et les adultes des lésions au niveau des membres inférieurs.

Les lésions prédominent au niveau des membres. Ce constat avait été fait par d'autres auteurs [3, 5]. Cela est dû à l'absence d'habitacle qui peut protéger les utilisateurs de cyclomoteur [5]. La chute d'un deux-roues motorisé peut entrainer une glissade qui occasionne des plaies et brûlures par frottement et se terminer par un choc contre un obstacle (trottoir, véhicule, rambarde de sécurité) qui va provoquer des lésions traumatiques secondaires. Cela explique le nombre important de lésions cutanéo-muqueuses chez nos patients qui ne portaient pas de tenue de protection le plus souvent. Dans les accidents de cyclomoteurs, les lésions les plus fréquentes sont celles des parties molles suivies des fractures [7]. Mais les lésions du tronc (thorax et abdomen) et de la tête sont les plus graves. En effet, dans notre série sur les 6 patients décédés, 5 présentaient des lésions de ces parties du corps. Dans les traumatismes du thorax, les lésions se font par compression. Elles siègent en regard du point d′impact au niveau pariétal et des structures directement sous-jacentes [8]. L′énergie cinétique au moment du traumatisme est le principal déterminant de la gravité des lésions. Les lésions d'organes intra-abdominaux se font par écrasement ou éclatement. Il s'agit essentiellement de perforations du tube digestif, d’éclatement d'organes pleins, de lacérations mésentériques par hyperpression directe ou de lésions vasculaires. La sévérité des lésions intra-abdominales augmente avec l’énergie du traumatisme [9]. La projection du patient sur le sol peut entraîner des traumatismes cranio-encéphaliques dont la gravité dépend de la vitesse de projection de l'accidenté sur le sol et du port ou non de casque. Le traumatisme du cerveau peut aussi être consécutif à la décélération brutale. Chaque mécanisme cause différents types de lésions allant de la commotion cérébrale à un traumatisme crânien mortel.

Les accidents de cyclomoteur sont également à l'origine de traumatisme à cinétique élevée et constituent des situations à risque de fracture du bassin. Ainsi la projection puis le glissement du bassin sur le réservoir peut entrainer une fracture en « livre ouvert » [10]. Tous le cas de fracture de bassin dans notre série sont dus à un traumatisme à haute cinétique. Nous n'avons pas observé de lésions secondaires à un glissement du bassin sur le réservoir car nos patients conduisaient des cyclomoteurs de petite cylindrée avec de petit réservoir.

## Conclusion

Les accidents de cyclomoteur constituent un véritable problème de santé publique. L'incidence dans nos régions ne fera qu'augmenter si les mesures correctrices ne sont pas appliquées [1]. La population jeune active est la plus intéressée. La chute de moto est le mécanisme le plus fréquent dans les petites agglomérations africaines. Les membres sont les plus touchés mais les lésions du tronc et de la tête sont les plus graves. Si ces accidents ne sont pas mortels ils sont responsables d'un important handicap physique au niveau de la population avec des retentissements socio-économiques liés à l'arrêt des activités génératrices de revenus mais aussi aux soins médicaux prolongés et chers.
